# Mixed-Sandwich Titanium(III) Qubits on Au(111): Electron Delocalization Ruled by Molecular Packing

**DOI:** 10.1021/acs.nanolett.2c03161

**Published:** 2022-10-18

**Authors:** Matteo Briganti, Giulia Serrano, Lorenzo Poggini, Andrea Luigi Sorrentino, Brunetto Cortigiani, Luana Carol de Camargo, Jaísa Fernandes Soares, Alessandro Motta, Andrea Caneschi, Matteo Mannini, Federico Totti, Roberta Sessoli

**Affiliations:** † Department of Chemistry “U. Schiff” (DICUS) and INSTM Research Unit, University of Florence, Via della Lastruccia 3-13, 50019 Sesto Fiorentino (FI), Italy; ‡ Department of Chemistry, Federal University of Parana, Centro Politecnico, Jardim das Americas, 81530-900 Curitiba, PR Brazil; § Department of Industrial Engineering (DIEF) and INSTM Research Unit, University of Florence, Via di Santa Marta, 3, 50139 Florence, Italy; ∥ Institute for Chemistry of OrganoMetallic Compounds (ICCOM-CNR), Via Madonna del Piano, 50019 Sesto Fiorentino (FI) Italy; ⊥ “La Sapienza” and INSTM Research Unit, University of Rome, Piazzale Aldo Moro 5, 00185 Rome, Italy

**Keywords:** scanning tunneling microscopy, X-ray photoelectron spectroscopy, density functional theory, molecular packing, organometallic sandwich compounds, molecule/surface interactions

## Abstract

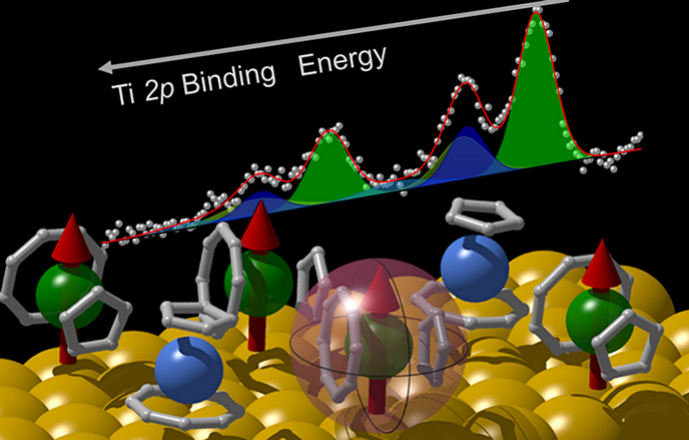

Organometallic sandwich complexes are versatile molecular systems that have been recently employed for single-molecule manipulation and spin sensing experiments. Among related organometallic compounds, the mixed-sandwich *S* = 1/2 complex (η^8^-cyclooctatetraene)(η^5^-cyclopentadienyl)titanium, here [CpTi(cot)], has attracted interest as a spin qubit because of the long coherence time. Here the structural and chemical properties of [CpTi(cot)] on Au(111) are investigated at the monolayer level by experimental and computational methods. Scanning tunneling microscopy suggests that adsorption occurs in two molecular orientations, lying and standing, with a 3:1 ratio. XPS data evidence that a fraction of the molecules undergo partial electron transfer to gold, while our computational analysis suggests that only the standing molecules experience charge delocalization toward the surface. Such a phenomenon depends on intermolecular interactions that stabilize the molecular packing in the monolayer. This orientation-dependent molecule–surface hybridization opens exciting perspectives for selective control of the molecule–substrate spin delocalization in hybrid interfaces.

Organometallic sandwich complexes hosting a transition metal (TM) or lanthanide ion show magnetic properties of great interest for spintronics and quantum computing.^1−5^ Metallocene derivatives containing dysprosium(III), for example, present single molecule magnet (SMM) behavior, i.e., magnetic memory at the molecular level up to record temperatures in the liquid nitrogen range.^6−8^ Cyclopentadienyl (Cp) ligands have also been employed to stabilize mixed valence Ln^II^–Ln^III^ dimeric SMMs exhibiting strong intramolecular ferromagnetic interaction and giant magnetic coercivity.^9^ Organometallic sandwich complexes are also an exciting playground for the on-surface realization of magnetic nanowires, which hold potential for spintronics applications.^10−12^ The easy manipulation of TM sandwich complexes by local probes has been used to localize spins on surfaces with atomic precision; this has been achieved by functionalizing a scanning tunneling microscope (STM) tip with a molecular unit.^13−15^ Inelastic electron tunneling microscopy measurements performed on nickelocene showed the signature of vibronic spin excitations, opening a route for manipulating the magnetic properties of individual molecules.^16^

Furthermore, the noticeable spin coherence time of some *3d* metal sandwich complexes makes them appealing for realizing molecular qubits.^17,18^ The confinement of these highly coherent systems on the surface is particularly attractive due to the most recent advances in STM experiments, where microwave fields coupled to the tunneling junction permitted both the resolution of electron spin resonance (ESR) signatures of magnetic atoms and molecules^19,20^ and control of their spin coherent properties.^21,22^

Recently, an *S* = 1/2 organometallic sandwich, the (η^8^-cyclooctatetraene)(η^5^-cyclopentadienyl)titanium, hereafter [CpTi(cot)] (Figures 1a and S1), revealed a noticeable coherence time of about 34 μs in a frozen toluene solution.^17^ This value is remarkably high considering the number of hydrogen atoms near the paramagnetic center, a well-known source of spin decoherence through the nuclear spin diffusion mechanism.^17,23^ The rich chemistry of these sandwich compounds also opens the possibility of realizing multiqubit systems to implement quantum gates.^18^ At variance with other molecular spin qubits such as copper or vanadyl porphyrins and phthalocyanine,^24−28^ the unpaired electron is located in a nonbonding d_*z*^2^_ orbital that is more prone to hybridization with the substrate and therefore highly interesting for local spin sensing experiments.^14,29−31^ To the best of our knowledge, no reports on the surface deposition of d^1^ sandwich complexes are available in the literature.

**Figure 1:**
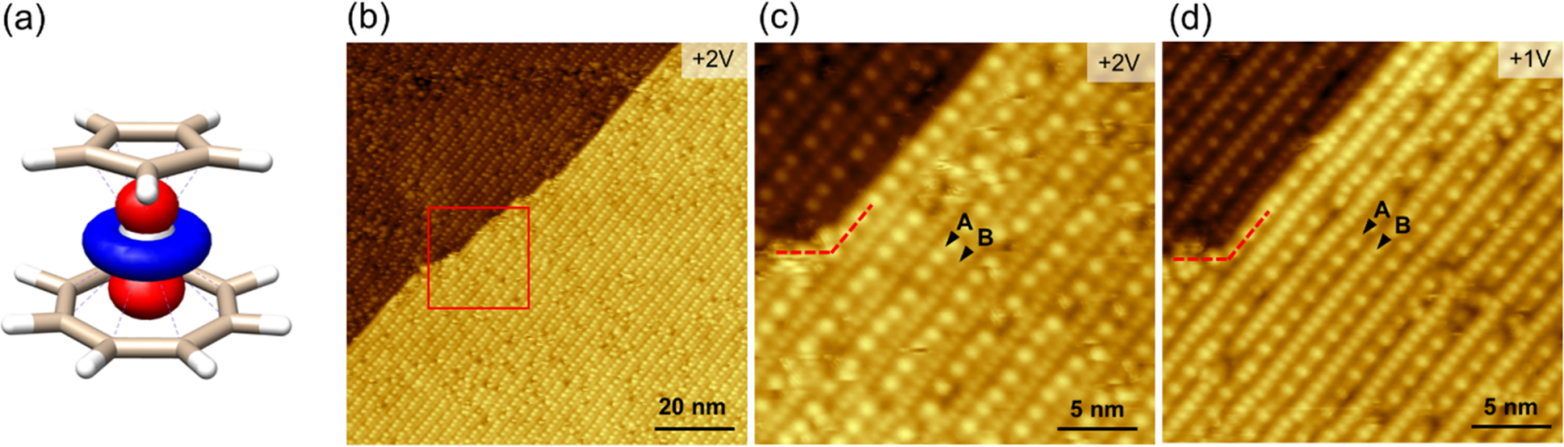
(a) Structure of the [CpTi(cot)] complex (white, hydrogen; light brown, carbon; gray, titanium) with the plot of the computed SOMO d_*z*^2^_ orbital. The orbital surfaces are drawn for a value of 0.05 e bohr^–3^. (b) STM image of a monolayer of [CpTi(cot)] on Au(111), *V*_b_= +2 V, *I*_t_ = 20pA. (c, d) Close-up view of the area marked in panel b. Empty state STM images recorded in constant current mode, *I*_t_ = 20 pA, with a bias voltage, *V*_b_, of +2 V (c) and +1 V (d). Dotted lines mark the step edge as a guide to the eye; black triangles indicate two bright rows (A and B) showing different STM height contrasts and periodicity.

Here we studied monolayer deposits of [CpTi(cot)] molecules on Au(111) surface by employing STM, X-ray and ultraviolet photoelectron spectroscopies (XPS and UPS), and density functional theory (DFT). The experimental STM images and the simulated adsorption geometry evidence that when these molecules pack on Au(111), they adopt two orientations to form a complex molecular packing. The adsorption potential energy surface (PES) was investigated theoretically to resolve the nontrivial and bias-dependent STM contrast. Calculations reveal that molecular packing governs the electron and spin delocalization of the molecular units, causing a variable extent of electron transfer from the molecule to the surface. An almost complete transfer of the Ti^III^ unpaired electron is computed for one specific adsorption conformation inside the complex pattern, in agreement with spectroscopic results. In this respect, the behavior of [CpTi(cot)] is significantly different from that of already investigated metallocenes such as ferrocene or nickelocene.^13,14,16,32,33^

The deposition of [CpTi(cot)] on the Au(111) surface, from here on [CpTi(cot)]@Au, results in a uniform and compact molecular monolayer shown in the large scale STM image of Figure 1b. Figure 1c,d shows the STM images at +2 and +1 V (empty states) of the area marked with a square in panel b. The molecular layer is characterized by round-shaped features organized in alternating bright and dark rows parallel to the Au(111) step edges. The STM contrast changes with the bias voltage (an even stronger bias dependence is observed at filled states and will be discussed afterward). The round features of the bright rows, labeled A and B, show a periodicity of 1.40 ±0.05 and 0.65 ± 0.05 nm, respectively (see height profiles in Figure S2). The dimension of round bright spots in the rows approximately matches the molecular dimensions, being 3.7 Å high and 5.5 Å wide (see Figure S1 and line profiles in Figure S2).

Similarly to literature results on other sandwich complexes such as nickelocene, molecular packing could not be directly deduced from the STM data.^32,33^ STM images and DFT calculations of nickelocene on copper and lead single crystals^32,33^ showed that bright and dark areas correspond to molecules adopting *standing* and *lying* orientations. We found that the less symmetric structure of [CpTi(cot)] as compared to bis(cyclopentadienyl) analogues, with different radial sizes of the cot and Cp ligands (Figures 1a and S1), further complicates the molecular arrangement and interpretation of the STM images. A comprehensive DFT characterization was performed to shed light on the molecular adsorption profile and properties of [CpTi(cot)]@Au. Several single-molecule optimizations were run at the periodic DFT level with periodic boundary conditions and Hubbard’s *U* correction on the *3d* orbitals (pDFT+*U*; see the Methods section in the Supporting Information) to scan the PES of the adsorption process. For the *standing* orientation, geometries with the cot (*standing*_*cot*_) and Cp (*standing*_*Cp*_) ligands in contact with the substrate were tested (see Figure 2). Different absorption sites, such as on-top, bridge, and fcc (Figure S3), were tested for all molecular orientations. For all orientations, the computed adsorption PES is essentially flat, with adsorption energies differing less than 2 kcal/mol and the fcc as the most favored site (Table S1). Concerning the overall PES for the three conformations, the *standing*_*cot*_ was the most stable, while the *lying*_*Cp*_ and *standing*_*Cp*_ were 5.5 and 5.9 kcal/mol higher in energy, respectively (see Figure 2). These energy differences cannot inhibit orientation changes, making it hard to establish *a priori* the preferential conformation. These considerations and the uneven pattern of bright features in the STM images (Figures 1 and 3) indicate that the unit cell describing the molecular pattern should include both *standing* and *lying* molecules.

**Figure 2:**
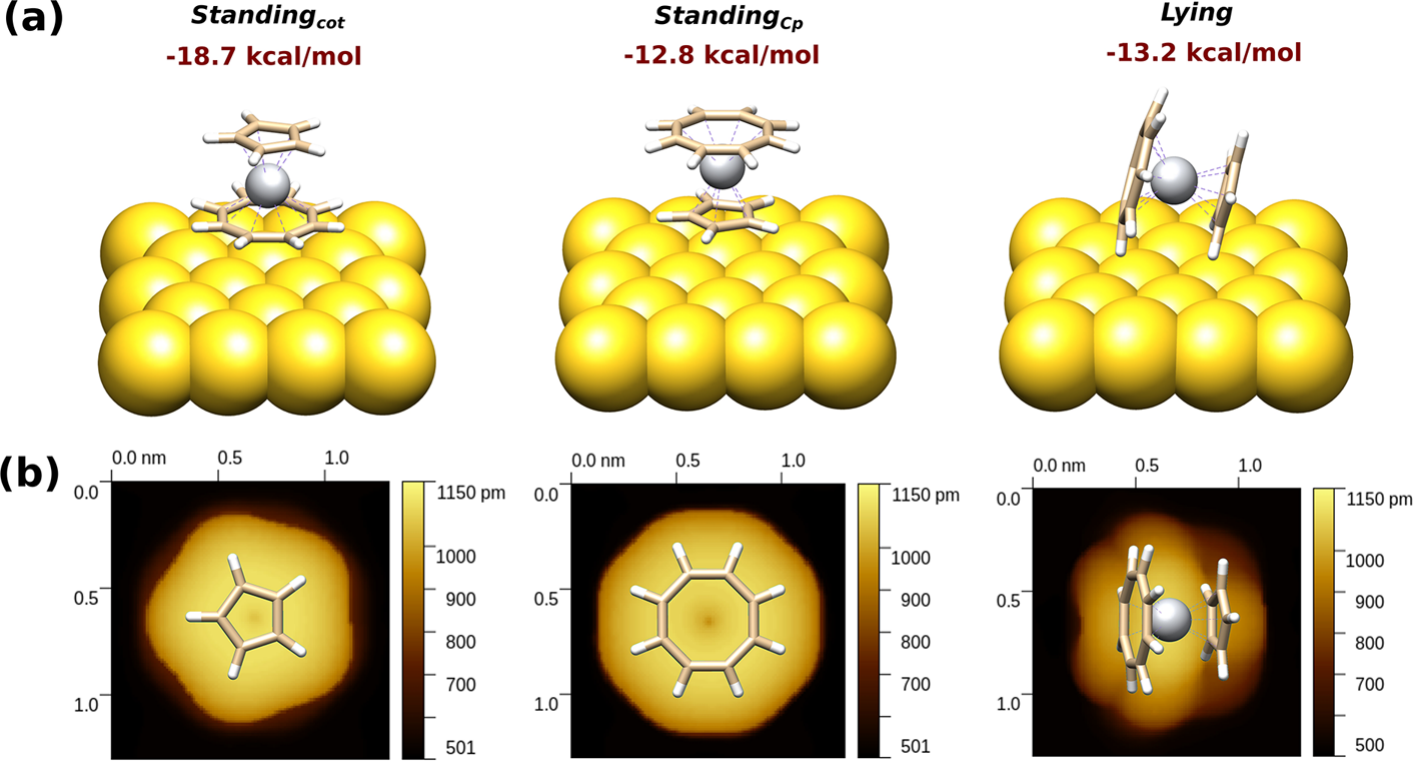
(a) Optimized structures and adsorption energies of isolated [CpTi(cot)] molecules on Au(111). (b) Simulated STM images (*V*_b_ = −2 V) for three distinct molecular orientations. Color code: white, hydrogen; light brown, carbon; gray, titanium; yellow, gold.

**Figure 3:**
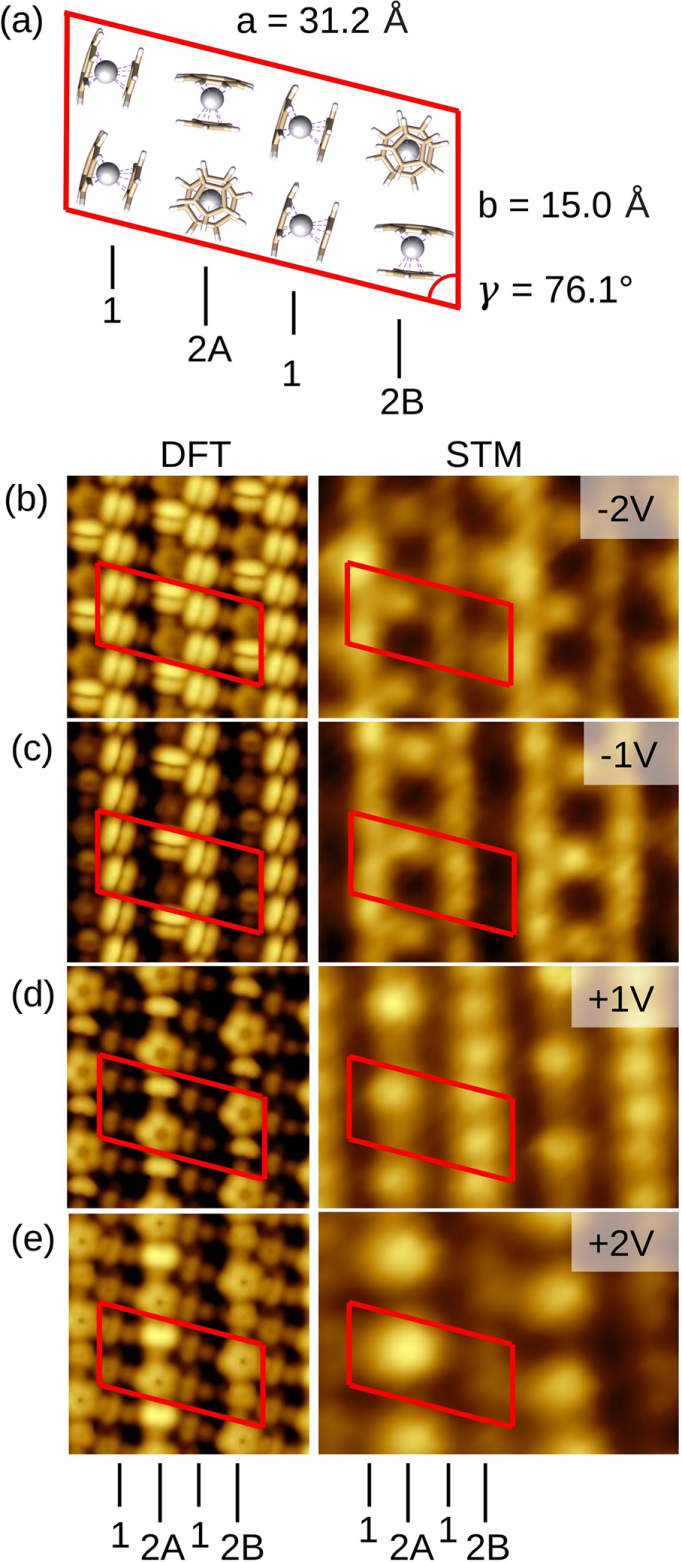
(a) Cell employed in the [CpTi(cot)]@Au optimization having six *lying* and two *standing*_*cot*_ molecules. Rows of molecules in the *lying* disposition are labeled 1, while rows presenting alternating *standing* and *lying* arrangements on the surface are labeled 2A and 2B. Panels b–e depict the simulated, left, and experimental STM images, right, of the [CpTi(cot)]@Au layer as a function of the bias voltage, *V*_b_. Simulations: 4.5 × 4.5 nm^2^, *V*_b_ = −2 V (b), −1 V (c), +2 V (d), +3 V (e). Experiment: 5.3 × 3.9 nm^2^, *I*_t_ = 20pA, *V*_b_ = −2 V (b), −1 V (c), +1 V (d), +2 V (e). Negative biases represent the sample’s filled states, and positive biases represent the empty ones. The unit cell and row labeling shown in (a) are marked as a guide to the eye.

The simulated STM images of the three [CpTi(cot)]@Au arrangements for the isolated molecule adsorbed on Au(111) sites appear as single rounded features in the two *standing* geometries and as a trilobed structure for the *lying* one (Figure 2b). Only slight differences were observed by varying the bias voltages at the filled or empty states (see Figures S4–S6). This contrasts with the experimental bias dependence, which further evolves when the filled states are sampled at a negative bias (Figure 3). In addition, no significant charge transfer from the isolated molecule to the surface is computed within either periodic slab (pDFT+U) or cluster (DFT) modeling (see the Methods section in the Supporting Information).

Because we could not reproduce the pronounced experimental bias dependence, we modeled the adsorbed monolayer of [CpTi(cot)] on Au(111) by considering unit cells with eight molecules having different combinations of adsorption orientations (see the Methods section in the Supporting Information). The comparison between the simulated STM images and the STM bias dependence (Figures 3 and S7) was taken as a benchmark to discriminate among the different simulated STM images.

The best match between the simulated and experimental STM data was obtained by considering unit cell dimensions of 31.2 and 15.0 Å, in agreement with the intermolecular distances observed in the crystal,^17^ and an angle of 76.1° between the unit lattice vectors. The unit cell consists of two *standing*_*cot*_ and six *lying* molecules (see Figure 3a). Molecules are arranged in two distinct types of rows: row 1 contains only *lying* molecules, while row 2 comprises alternating *lying* and *standing*_*cot*_ molecules. In the model, two nonequivalent rows of the second type, differing in the order of the alternating *standing* and *lying* molecules, were also included. They are labeled 2A and 2B to recall the two distinct bright rows of the STM image in Figure 1.

STM images on the fully optimized array (see the Methods section in the Supporting Information) were calculated by periodic DFT with Hubbard’s *U* correction (pDFT+*U*) at different biases. An excellent agreement with experimental STM images was observed (see Figure 3b–e). At −2 V, the bright features (Figure 3b) correspond to *lying* molecules in rows 1 and 2, while dark areas correspond to the *standing*_*cot*_ molecules in rows 2. At −1 V (Figure 3c), the lying molecule in row 2B completely and selectively loses its brightness, leading to a completely dark row. At positive biases, the STM contrast is almost completely reversed: at +1 V, *lying* molecules in row 1 appear as dark areas, while in rows 2 they appear as bright spots, the brightest being the *lying* molecules in row 2A (Figure 3d). This trend further enhances at +2 V, in agreement with the large-scale images in Figure 1. An energy shift of 1 V between the experimental and computed bias voltages was detected at positive biases (see caption in Figure 3); this difference probably comes from upward energy shifts of the virtual/empty orbitals resulting from the employed DFT method.^34^ Additionally, a mismatch in the cell dimensions of about 12% is within the experimental error and can be due to the enforced commensurability between substrate and adsorbate lattices.^35^ Interestingly, all attempts to reproduce the STM images using the alternating *standing-lying* arrangement typical of metallocenes^32,36^ produced a bias dependence incompatible with our experimental results, as shown in Figure S7.

A molecular layer superstructure similar to that shown in Figure 3 was observed in the STM conductance maps of nickelocene on Pb(111) and ascribed to different magnetic anisotropy energies (MAE) of the molecules induced by the adsorption site.^33^ In that case, however, the applied STM bias voltages were up to a few millivolts; consequently, tiny variations of the orbital energy ladders could be monitored. In [CpTi(cot)], the superstructure’s bias dependence is observed at 3 orders of magnitude larger energies, leading to more pronounced electronic effects. Indeed, according to our calculations, *lying* molecules in the array show negligible to weak charge transfer to the substrate: the computed unpaired electron populations in the Ti *3d* orbitals are 1.18, 1.08, and 0.85 for the *lying* molecules in rows 1, 2A, and 2B, respectively. The different degree of charge and spin delocalization to the surface between the last two cases comes from the adsorption site: in row 2A, the *cot* ligand sits on top of a gold atom leading to a larger Ti–surface distance and, consequently, a more significant charge preservation. In row 2B, in turn, the cot ring is placed between two gold atoms, shortening the Ti–Au distance.

At variance with the isolated molecule calculations, a strong electron delocalization to the surface is computed for the *standing*_*cot*_ molecules in the array (type 2 rows). This phenomenon is shown in Figure S11, where a selective transfer of spin density from the *standing* molecules to the surface takes place. The DFT population analysis shows complete draining of the unpaired electron (0.01 unpaired electron on the Ti) toward the surface, leading to a formal +IV oxidation state (*3d*^0^ configuration) for the metal atom.

To support the hypothesis of selective oxidation of the *standing* molecules, the Ti *3d* and C *2p* projected density of states (PDOS) are reported in Figure S12. From their analysis, the peculiar bias dependence of the STM images can also be rationalized: (i) both the Ti *3d* and C *2p* orbitals contribute to the on/off bias dependence shown by the *lying* molecules; (ii) only the C *2p* orbitals contribute to the bias dependence of the *standing* molecules because the carbon electron density of the ligand shields the *3d* orbitals from the STM tip.

To gain further insight into the charge transfer occurring at the molecule–metal interface, the [CpTi(cot)]@Au layer was investigated by XPS and UPS photoelectron spectroscopies. The XPS core-level Ti *2p* and C *1s* spectra are reported in Figure 4. The semiquantitative elemental analysis (Table S2) shows that the total amount of carbon and titanium detected by XPS is in good agreement with the elemental composition of [CpTi(cot)], thus confirming that only intact molecules are assembled on the surface. Noticeably, the Ti *2p_3/2_* XPS region shows two distinct contributions at 455.2 eV (I) and 457.3 eV (II), with the respective Ti *2p_1/2_* spin–orbit (SO) components 6.1 eV away from the main Ti *2p_3/2_* peaks.^37^ According to the literature, the feature at the lowest binding energy, 455.2 eV, with a related shakeup feature at 457.0 eV, could be assigned to the Ti^III^ centers in the [CpTi(cot)] molecules.^38−40^ The presence of Ti^III^ is also supported by UPS results compared with the calculated DOS (see details in the Supporting Information and Figure S8). However, the XPS intensity of the feature at 457.0 eV exceeds that expected for a Ti^III^ shakeup feature. By fixing this intensity in the simulation procedure (cyano component) to that expected from the literature,^39^ we can evidence an additional contribution (peak II) centered at 457.3 eV and shown in orange, with its respective SO and shakeup satellites (Figure 4).

**Figure 4:**
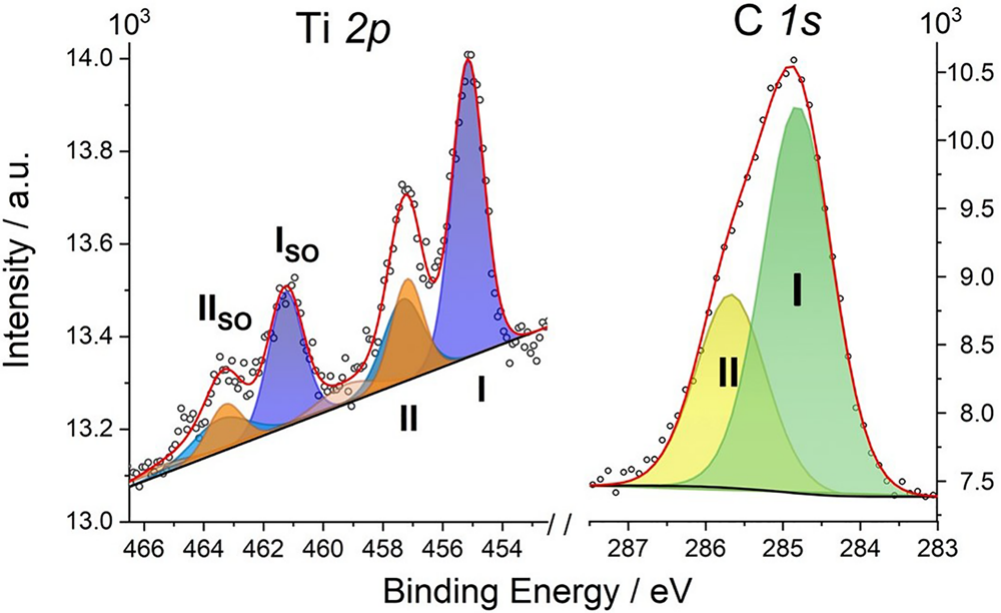
XPS core-level spectra of [CpTi(cot)] on the Au(111) surface in the Ti *2p* and C *1s* regions. Labels I and II indicate the main components of each spectral region, as discussed in the text. The spin–orbit components are labeled “SO”.

The area ratio of the different contributions provides further clues on the assignment of the XPS peaks. For the Ti *2p* region, the ratio between the blue and the orange areas is about 2.7 ± 0.1, which is in reasonable agreement with the ratio of the *lying* and *standing* molecules in the computed unit cell (Figure 3). This experimental result supports our hypothesis that only molecules in the standing orientation undergo a significant charge transfer to the surface, giving rise to the higher binding energy of peaks II and II(SO) for the Ti *2p* electrons.

Interestingly, the XPS C *1s* core level spectrum (Figure 4) also shows an asymmetric signal centered around 285.0 eV, in agreement with the characteristic carbon signal for a Cp ring in similar organometallic species deposited on metals.^37,41,42^ The asymmetry of this signal indicates two components, I and II, that are fitted respectively at 284.8 and 285.7 eV with a 2.4 ± 0.1 ratio. This proportion is intermediate between the fraction of carbon atoms in the cot and Cp rings, 1.6, and the ratio of the Ti *2p* components (see the Supporting Information for further details, Figure S10 and Table S4). This finding further supports the presence of two types of deposited molecules, with and without significant charge transfer to the surface.

Given the scarce literature reports about XPS signals from metallocenes and even less about mixed-sandwich complexes, a clear attribution of the observed XPS signals is not straightforward. Different environments and oxidation states of the Ti atom were then evaluated by DFT, as explained in the Supporting Information, to reproduce the XPS features. The computed binding energies are compared with the available literature data in Table S3. Changing the formal oxidation state from [(η^5^-Cp^s^)_2_Ti^II^] (this is a “titanocene” complex;^43,44^ see theoretical methods in the Supporting Information) to [CpTi^III^(cot)] and then to [CpTi^IV^(cht)], where cht^3–^ is the anion of cycloheptatriene, the binding energy increases stepwise of ca. 1 eV for each electron loss. On the basis of these results and considering that our average absolute error on the computed binding energies is ca. 3 eV, the experimental peak at 455.0 eV (computed at 452.1 eV) can be safely assigned to a Ti^III^ atom with its *2p_1/2_* component at 461.3 eV. While the presence of Ti^II^ can be definitively excluded, the same is not valid for Ti^IV^. The binding energy for [CpTi^IV^(cht)] has been determined to be 456.1 eV^37^ vs 453.1 eV in our calculations, suggesting that the XPS peaks for a positively charged [CpTi^IV^(cot)]^+^ species should shift further to even higher energies. This allows us to associate the minor components of the Ti *2p* (II and II_so_ in Figure 4) to an oxidized form, probably [CpTi^IV^(cot)]^+^, in agreement with the complete electron transfer toward the surface computed for the *standing* conformation.

Recalling that pDFT+*U* performed on isolated [CpTi(cot)]@Au in different orientations did not reveal any oxidation, the selective changing from Ti^III^ to Ti^IV^ in *standing*_*cot*_ can be likely ascribed to synergic packing/surface effects. To verify this hypothesis, two scenarios were considered: (i) the Au(111) surface was removed from the optimized array of the [CpTi(cot)]@Au monolayer; (ii) only the two *standing* molecules from the optimized array structure were left on the surface, while the six *lying* molecules were removed. In both cases, no significant oxidation process occurs: the complete draining of the unpaired electron from the *standing* molecules is found only when the initially modeled monolayer array of [CpTi(cot)]@Au is computed (see Figure S11). This finding suggests that the selective oxidation and loss of the unpaired electron observed for the *standing*_*cot*_ molecules can only occur due to intermolecular interactions inside the monolayer, which can stabilize the higher titanium oxidation state. The process results from the synergy with the metallic surface, which drains the unpaired electrons.

In conclusion, a highly promising *S* = 1/2 molecular qubit^17^ has been deposited on Au(111) as the first step toward single-molecule addressing. The adsorption process, investigated with a combined experimental and theoretical approach, indicates that the asymmetric [CpTi(cot)] molecule assembles in a complex monolayer arrangement with a 3:1 ratio of *lying* and *standing* molecules that is unprecedented for organometallic sandwich compounds on surfaces. The presence of an unpaired electron in the d_*z*^2^_ orbital, unusual for d^1^/d^9^ molecules, results in a multifaceted and challenging scenario: a pronounced bias dependence of the STM contrast and different oxidation states for titanium, the latter suggested by XPS data. This puzzling picture was rationalized by multilevel computational analysis showing that an oxidation process selectively takes place on the surface only for the *standing*_*cot*_ molecules whose unpaired electron is completely transferred to the surface. Conversely, the spin density is substantially preserved for the *lying* molecules, so rationalizing the experimental evidence for both Ti^IV^ and Ti^III^ in the XPS spectrum. The different adsorption sites in the periodic supercell provide a nontrivial variation of the STM images as a function of the applied bias, in agreement with experimental findings. It is worth stressing that due to the synergic effect of molecular packing and molecule–surface interactions, the selective oxidation of the *standing*_*cot*_ molecules only takes place if the dense monolayer is modeled. These results suggest the possibility of selective control of spin delocalization in molecular monolayers through chemically tailored intermolecular interactions. They also identify [CpTi(cot)] as an interesting molecular system to probe spin coherence and distribution in densely packed molecular layers by ESR-STM experiments^20^ as well as to realize hybrid molecular/superconductor interfaces where through-ligand coupled states in molecular networks may trigger topological quantum properties.^45^

## Supporting Information Available

STM experimental images and profiles, simulated STM images by pDFT, UPS, and XPS data (experimental and simulated by DFT methods), computed spin densities, and DOS (PDF)

## References

[ref1] Gould C. A.; McClain K. R.; Yu J. M.; Groshens T. J.; Furche F.; Harvey B. G.; Long J. R. Synthesis and Magnetism of Neutral, Linear Metallocene Complexes of Terbium(II) and Dysprosium(II). J. Am. Chem. Soc. 2019, 141, 12967–12973. 10.1021/jacs.9b05816. 31375028

[ref2] Bunting P. C.; Atanasov M.; Damgaard-Møller E.; Perfetti M.; Crassee I.; Orlita M.; Overgaard J.; van Slageren J.; Neese F.; Long J. R. A Linear Cobalt(II) Complex with Maximal Orbital Angular Momentum from a Non-Aufbau Ground State. Science 2018, 362, eaat7319 10.1126/science.aat7319. 30442763

[ref3] Zadrozny J. M.; Xiao D. J.; Atanasov M.; Long G. J.; Grandjean F.; Neese F.; Long J. R. Magnetic Blocking in a Linear Iron(I) Complex. Nat. Chem. 2013, 5, 577–581. 10.1038/nchem.1630. 23787747

[ref4] Ariciu A.-M.; Woen D. H.; Huh D. N.; Nodaraki L. E.; Kostopoulos A. K.; Goodwin C. A. P.; Chilton N. F.; McInnes E. J. L.; Winpenny R. E. P.; Evans W. J.; Tuna F. Engineering Electronic Structure to Prolong Relaxation Times in Molecular Qubits by Minimising Orbital Angular Momentum. Nat. Commun. 2019, 10, 3330 10.1038/s41467-019-11309-3. 31350411 PMC6659626

[ref5] Ding M.; Hickey A. K.; Pink M.; Telser J.; Tierney D. L.; Amoza M.; Rouzières M.; Ozumerzifon T. J.; Hoffert W. A.; Shores M. P.; Ruiz E.; Clérac R.; Smith J. M. Magnetization Slow Dynamics in Ferrocenium Complexes. Chem. - Eur. J. 2019, 25, 10625–10632. 10.1002/chem.201900799. 31066934

[ref6] Goodwin C. A. P.; Ortu F.; Reta D.; Chilton N. F.; Mills D. P. Molecular Magnetic Hysteresis at 60 K in Dysprosocenium. Nature 2017, 548, 439–442. 10.1038/nature23447. 28836589

[ref7] Guo F.-S.; Day B. M.; Chen Y.-C.; Tong M.-L.; Mansikkamäki A.; Layfield R. A. A Dysprosium Metallocene Single-Molecule Magnet Functioning at the Axial Limit. Angew. Chemie Int. Ed. 2017, 56, 11445–11449. 10.1002/anie.201705426. 28586163

[ref8] Pedrini A.; Poggini L.; Tudisco C.; Torelli M.; Giuffrida A. E.; Bertani F.; Cimatti I.; Otero E.; Ohresser P.; Sainctavit P.; Suman M.; Condorelli G. G.; Mannini M.; Dalcanale E. Self-Assembly of TbPc_2_ Single-Molecule Magnets on Surface through Multiple Hydrogen Bonding. Small 2018, 14, 1702572 10.1002/smll.201702572. 29226595

[ref9] Gould C. A.; McClain K. R.; Reta D.; Kragskow J. G. C.; Marchiori D. A.; Lachman E.; Choi E.-S.; Analytis J. G.; Britt R. D.; Chilton N. F.; Harvey B. G.; Long J. R. Ultrahard Magnetism from Mixed-Valence Dilanthanide Complexes with Metal-Metal Bonding. Science 2022, 375, 198–202. 10.1126/science.abl5470. 35025637

[ref10] Huttmann F.; Schleheck N.; Atodiresei N.; Michely T. On-Surface Synthesis of Sandwich Molecular Nanowires on Graphene. J. Am. Chem. Soc. 2017, 139, 9895–9900. 10.1021/jacs.7b03381. 28682606

[ref11] Huttmann F.; Rothenbach N.; Kraus S.; Ollefs K.; Arruda L. M.; Bernien M.; Thonig D.; Delin A.; Fransson J.; Kummer K.; Brookes N. B.; Eriksson O.; Kuch W.; Michely T.; Wende H. Europium Cyclooctatetraene Nanowire Carpets: A Low-Dimensional, Organometallic, and Ferromagnetic Insulator. J. Phys. Chem. Lett. 2019, 10, 911–917. 10.1021/acs.jpclett.8b03711. 30717591

[ref12] Kraus S.; Herman A.; Huttmann F.; Bianchi M.; Stan R. M.; Holt A. J.; Tsukamoto S.; Rothenbach N.; Ollefs K.; Dreiser J.; Bischof K.; Wende H.; Hofmann P.; Atodiresei N.; Michely T. Uniaxially Aligned 1D Sandwich-Molecular Wires: Electronic Structure and Magnetism. J. Phys. Chem. C 2022, 126, 3140–3150. 10.1021/acs.jpcc.1c10625.

[ref13] Ormaza M.; Bachellier N.; Faraggi M. N.; Verlhac B.; Abufager P.; Ohresser P.; Joly L.; Romeo M.; Scheurer F.; Bocquet M.-L.; Lorente N.; Limot L. Efficient Spin-Flip Excitation of a Nickelocene Molecule. Nano Lett. 2017, 17, 1877–1882. 10.1021/acs.nanolett.6b05204. 28199115

[ref14] Verlhac B.; Bachellier N.; Garnier L.; Ormaza M.; Abufager P.; Robles R.; Bocquet M.-L.; Ternes M.; Lorente N.; Limot L. Atomic-Scale Spin Sensing with a Single Molecule at the Apex of a Scanning Tunneling Microscope. Science 2019, 366, 623–627. 10.1126/science.aax8222. 31672895

[ref15] Czap G.; Wagner P. J.; Xue F.; Gu L.; Li J.; Yao J.; Wu R.; Ho W. Probing and Imaging Spin Interactions with a Magnetic Single-Molecule Sensor. Science 2019, 364, 670–673. 10.1126/science.aaw7505. 31097665

[ref16] Bachellier N.; Verlhac B.; Garnier L.; Zaldívar J.; Rubio-Verdú C.; Abufager P.; Ormaza M.; Choi D. J.; Bocquet M. L.; Pascual J. I.; Lorente N.; Limot L. Vibron-Assisted Spin Excitation in a Magnetically Anisotropic Molecule. Nat. Commun. 2020, 11, 1619 10.1038/s41467-020-15266-0. 32238814 PMC7113279

[ref17] Camargo L. C.; Briganti M.; Santana F. S.; Stinghen D.; Ribeiro R. R.; Nunes G. G.; Soares J. F.; Salvadori E.; Chiesa M.; Benci S.; Torre R.; Sorace L.; Totti F.; Sessoli R. Exploring the Organometallic Route to Molecular Spin Qubits: The [CpTi(Cot)] Case. Angew. Chemie Int. Ed. 2021, 60, 2588–2593. 10.1002/anie.202009634. 33051985

[ref18] von Kugelgen S.; Krzyaniak M. D.; Gu M.; Puggioni D.; Rondinelli J. M.; Wasielewski M. R.; Freedman D. E. Spectral Addressability in a Modular Two Qubit System. J. Am. Chem. Soc. 2021, 143, 8069–8077. 10.1021/jacs.1c02417. 34014650

[ref19] Baumann S.; Paul W.; Choi T.; Lutz C. P.; Ardavan A.; Heinrich A. J. Electron Paramagnetic Resonance of Individual Atoms on a Surface. Science 2015, 350, 417–420. 10.1126/science.aac8703. 26494753

[ref20] Zhang X.; Wolf C.; Wang Y.; Aubin H.; Bilgeri T.; Willke P.; Heinrich A. J.; Choi T. Electron Spin Resonance of Single Iron Phthalocyanine Molecules and Role of Their Non-Localized Spins in Magnetic Interactions. Nat. Chem. 2022, 14, 59–65. 10.1038/s41557-021-00827-7. 34764471

[ref21] Willke P.; Bilgeri T.; Zhang X.; Wang Y.; Wolf C.; Aubin H.; Heinrich A.; Choi T. Coherent Spin Control of Single Molecules on a Surface. ACS Nano 2021, 15, 17959–17965. 10.1021/acsnano.1c06394. 34767351

[ref22] Yang K.; Paul W.; Phark S.-H.; Willke P.; Bae Y.; Choi T.; Esat T.; Ardavan A.; Heinrich A. J.; Lutz C. P. Coherent Spin Manipulation of Individual Atoms on a Surface. Science 2019, 366, 509–512. 10.1126/science.aay6779. 31649202

[ref23] Canarie E. R.; Jahn S. M.; Stoll S. Quantitative Structure-Based Prediction of Electron Spin Decoherence in Organic Radicals. J. Phys. Chem. Lett. 2020, 11, 3396–3400. 10.1021/acs.jpclett.0c00768. 32282218 PMC7654569

[ref24] Atzori M.; Morra E.; Tesi L.; Albino A.; Chiesa M.; Sorace L.; Sessoli R. Quantum Coherence Times Enhancement in Vanadium(IV)-Based Potential Molecular Qubits: The Key Role of the Vanadyl Moiety. J. Am. Chem. Soc. 2016, 138, 11234–11244. 10.1021/jacs.6b05574. 27517709

[ref25] Yu C. J.; Graham M. J.; Zadrozny J. M.; Niklas J.; Krzyaniak M. D.; Wasielewski M. R.; Poluektov O. G.; Freedman D. E. Long Coherence Times in Nuclear Spin-Free Vanadyl Qubits. J. Am. Chem. Soc. 2016, 138, 14678–14685. 10.1021/jacs.6b08467. 27797487

[ref26] Zadrozny J. M.; Niklas J.; Poluektov O. G.; Freedman D. E. Millisecond Coherence Time in a Tunable Molecular Electronic Spin Qubit. ACS Cent. Sci. 2015, 1, 488–492. 10.1021/acscentsci.5b00338. 27163013 PMC4827467

[ref27] Bader K.; Dengler D.; Lenz S.; Endeward B.; Jiang S.-D.; Neugebauer P.; van Slageren J. Room Temperature Quantum Coherence in a Potential Molecular Qubit. Nat. Commun. 2014, 5, 5304 10.1038/ncomms6304. 25328006

[ref28] Bader K.; Winkler M.; Van Slageren J. Tuning of Molecular Qubits: Very Long Coherence and Spin-Lattice Relaxation Times. Chem. Commun. 2016, 52, 3623–3626. 10.1039/C6CC00300A. 26854001

[ref29] Hatter N.; Heinrich B. W.; Ruby M.; Pascual J. I.; Franke K. J. Magnetic Anisotropy in Shiba Bound States across a Quantum Phase Transition. Nat. Commun. 2015, 6, 1–6. 10.1038/ncomms9988. PMC4674822 26603561

[ref30] Hatter N.; Heinrich B. W.; Rolf D.; Franke K. J. Scaling of Yu-Shiba-Rusinov Energies in the Weak-Coupling Kondo Regime. Nat. Commun. 2017, 8, 2016 10.1038/s41467-017-02277-7. 29222411 PMC5722882

[ref31] Malavolti L.; Briganti M.; Hänze M.; Serrano G.; Cimatti I.; McMurtrie G.; Otero E.; Ohresser P.; Totti F.; Mannini M.; Sessoli R.; Loth S. Tunable Spin-Superconductor Coupling of Spin 1/2 Vanadyl-Phthalocyanine Molecules. Nano Lett. 2018, 18, 7955–7961. 10.1021/acs.nanolett.8b03921. 30452271

[ref32] Bachellier N.; Ormaza M.; Faraggi M.; Verlhac B.; Vérot M.; Le Bahers T.; Bocquet M.-L.; Limot L. Unveiling Nickelocene Bonding to a Noble Metal Surface. Phys. Rev. B 2016, 93, 195403 10.1103/PhysRevB.93.195403.

[ref33] Mier C.; Verlhac B.; Garnier L.; Robles R.; Limot L.; Lorente N.; Choi D.-J. Superconducting Scanning Tunneling Microscope Tip to Reveal Sub-Millielectronvolt Magnetic Energy Variations on Surfaces. J. Phys. Chem. Lett. 2021, 12, 2983–2989. 10.1021/acs.jpclett.1c00328. 33730501

[ref34] Van Meer R.; Gritsenko O. V.; Baerends E. J. Physical Meaning of Virtual Kohn-Sham Orbitals and Orbital Energies: An Ideal Basis for the Description of Molecular Excitations. J. Chem. Theory Comput. 2014, 10, 4432–4441. 10.1021/ct500727c. 26588140

[ref35] Hofmann O. T.; Zojer E.; Hörmann L.; Jeindl A.; Maurer R. J. First-Principles Calculations of Hybrid Inorganic-Organic Interfaces: From State-of-the-Art to Best Practice. Phys. Chem. Chem. Phys. 2021, 23, 8132–8180. 10.1039/D0CP06605B. 33875987 PMC8237233

[ref36] Ormaza M.; Abufager P.; Bachellier N.; Robles R.; Verot M.; Le Bahers T.; Bocquet M.-L.; Lorente N.; Limot L. Assembly of Ferrocene Molecules on Metal Surfaces Revisited. J. Phys. Chem. Lett. 2015, 6, 395–400. 10.1021/jz5026118. 26261954

[ref37] Groenenboom C. J.; Sawatzky G.; de Liefde Meijer H. J.; Jellinek F. Electron Spectroscopy of Some Cyclopentadienylcycloheptatrienylmetal Compounds. J. Organomet. Chem. 1974, 76, C4–C6. 10.1016/S0022-328X(00)90324-3.

[ref38] Jaeger D.; Patscheider J. Single Crystalline Oxygen-Free Titanium Nitride by XPS. Surf. Sci. Spectra 2013, 20 (1), 1–8. 10.1116/11.20121107.

[ref39] Jaeger D.; Patscheider J. A Complete and Self-Consistent Evaluation of XPS Spectra of TiN. J. Electron Spectrosc. Relat. Phenom. 2012, 185, 523–534. 10.1016/j.elspec.2012.10.011.

[ref40] Porte L.; Roux L.; Hanus J. Vacancy Effects in the X-Ray Photoelectron Spectra of TiNx. Phys. Rev. B 1983, 28, 3214–3224. 10.1103/PhysRevB.28.3214.

[ref41] Paul R.; Reifenberger R. G.; Fisher T. S.; Zemlyanov D. Y. Atomic Layer Deposition of FeO on Pt(111) by Ferrocene Adsorption and Oxidation. Chem. Mater. 2015, 27, 5915–5924. 10.1021/acs.chemmater.5b01778.

[ref42] Woodbridge C. M.; Pugmire D. L.; Johnson R. C.; Boag N. M.; Langell M. A. HREELS and XPS Studies of Ferrocene on Ag(100). J. Phys. Chem. B 2000, 104, 3085–3093. 10.1021/jp993235+.

[ref43] Luo Y.; Ohno K. Computational Study of Titanocene-Catalyzed Dehydrocoupling of the Adduct Me2NH·BH3: An Intramolecular, Stepwise Mechanism. Organometallics 2007, 26, 3597–3600. 10.1021/om7003892.

[ref44] Hitchcock P. B.; Kerton F. M.; Lawless G. A. The Elusive Titanocene. J. Am. Chem. Soc. 1998, 120, 10264–10265. 10.1021/ja981934e.

[ref45] Pawlak R.; Hoffman S.; Klinovaja J.; Loss D.; Meyer E. Majorana Fermions in Magnetic Chains. Prog. Part. Nucl. Phys. 2019, 107, 1–19. 10.1016/j.ppnp.2019.04.004.

