# Supplementary material for: Mixed-Sandwich Titanium(III) Qubits on Au(111): Electron Delocalization Ruled by Molecular Packing
Source: Nano Lett. 2022 Oct 18;22(21):8626–32. doi: 10.1021/acs.nanolett.2c03161 (PMC9650780; doi:10.1021/acs.nanolett.2c03161)
Supplement: Supplementary file 1 — nl2c03161_si_001.pdf [file nl2c03161_si_001.pdf]

Supporting Information for

Mixed-sandwich titanium(III) qubits on Au(111): electron delocalization ruled by molecular packing

Matteo Briganti,[1,2†] Giulia Serrano,[3*†] Lorenzo Poggini,[1,4*] Andrea Luigi Sorrentino,[1,3] Brunetto Cortigiani,[1] Luana Carol de Camargo,[2] Jaísa Fernandes Soares,[2] Alessandro Motta,[5] Andrea Caneschi,[3] Matteo Mannini,[1] Federico Totti,[1*] Roberta Sessoli[1*]*

1) Department of Chemistry "U. Schiff" (DICUS) and INSTM Research Unit, University of Florence, Via della Lastruccia 3-13, 50019 Sesto Fiorentino (FI), Italy

2) Department of Chemistry, Federal University of Parana, Centro Politecnico, Jardim das Americas, 81530-900 Curitiba-PR, Brazil

3) Department of Industrial Engineering (DIEF) and INSTM Research Unit, University of Florence, Via di Santa Marta, 3, 50139, Florence, Italy

4) Institute for Chemistry of OrganoMetallic Compounds (ICCOM-CNR), Via Madonna del Piano, 50019 Sesto Fiorentino (FI), Italy

5) University of Rome "La Sapienza" and INSTM Research Unit, Piazzale Aldo Moro 5, 00185 Rome, Italy.

†These authors contributed equally to this manuscript

Table of Contents

1 – Methods

1.1 – Experimental Methods……………………………………………3

1.2 – Theoretical Methods…………………………………...……4

2 – Additional Information

2.1 – Supporting Figures and Tables ……..……..……………………7

2.2 – UPS Analysis……………………………………………………...12

2.3 – Simulation of XPS Results…………………………...…..…13

2.4 – Simulated PDOS……..…………………………………………17

3 References 20

1 - Methods

1.1 - Experimental Methods

Synthesis. [CpTi(cot)] was synthetized and characterized according to the procedure reported by de Camargo et al.,[1] based on the reaction of [Cp_2TiCl_2] and cyclooctatetraene with n-butyl lithium in tetrahydrofuran. To obtain high purity crystalline samples for surface studies, the product was sublimed at *ca* 130 °C/10^{-3} mm Hg and then recrystallized from saturated toluene solutions at – 20°C. Due to the high molecular sensitivity to the air, the crystals were isolated and handled under inert atmosphere and stored in sealed glass tubes. To perform deposition in ultra-high vacuum (UHV), the crucible for molecular sublimation was filled in a dry N_2 glove box, and then transferred in static vacuum to the deposition chamber.

Monolayer samples preparation. The Au(111) surface was prepared in UHV by sputtering cycles with Ar ions (1.5 keV energy) and subsequent annealing at 770 K. Surface cleanliness and reconstruction were controlled by XPS and STM measurements after preparation. Molecules were deposited in UHV by exposing the Au(111) surface to the molecular flux with both substrate and crucible kept at RT (with a base pressure of $2x10^{-8}$mbar).

STM characterization. The STM characterization was carried out by an Omicron Variable-Temperature STM in vacuum connection with the molecular sublimation chamber. STM images of clean substrates were collected at room temperature, while samples with molecular deposits were measured at 30 K, to stabilize the molecules during the scanning.

XPS analysis. XPS analyses were performed with a micro-focused mono-chromatic Al Kα radiation source, hν = 1,486.7 eV (XR-MF + Focus 600, by SPECS), and a multichannel detector electron analyzer (SPECS Phoibos 150 1DLD). XPS measurements were recorded in normal emission and using a pass energy of 40 eV. The X-ray mono-chromatic beam was set at 54.44° with respect to the analyzer. XPS spectra were analyzed using the CasaXPS software and fitted using a Shirley

background. Single-peak components were deconvoluted by a mixed Gaussian and Lorentzian function (70/30). Spectra were calibrated using the Au *4f* component at 84.0 eV.

UPS analysis. UPS data were collected with a non-monochromatized gas discharge UV lamp (VG Scientific 22-101) using the He(II) line (40.8 eV). The analyzer pass energy was set to 10 eV, and a −30 V bias was applied to the sample. UPS spectra were measured at normal emission and calibrated to the Fermi energy of Au(111).

All the photoelectron experiments were performed at 150 K to avoid desorption of the molecular layer.

1.2 - Theoretical Methods

Periodic DFT simulations. The CP2K package was used[2–4] along with rVV10 non-local empirical dispersion corrections.[5] Norm-conserving Goedecker-Tetter-Hutter pseudopotentials[6] and a double zeta basis set with polarization functions (DZVP-MOLOPT-SR) were employed for all the atoms. The cell parameters were kept fixed throughout the optimizations. The plane-wave cut-off value was set to 400 Ry. The wavefunction convergence (EPS_SCF) was set to $1.0x10^{-7}$ Hartree, while the max force for the geometry optimization was set to $4.5x10^{-4}$ $bohr^{-1}$Hartree. A model orthorhombic cell of dimensions 17.31 Å × 14.99 Å × 30 Å was used throughout these optimizations. A monoclinic unit cell (31.206 Å × 14.991 Å × 30 Å, $\alpha = \beta = 90°$, and $\gamma = 76.102°$) was used to reproduce the experimental STM images of the adsorbed monolayer. To simulate the monolayer properties, eight [CpTi(cot)] molecules were set on an Au(111) surface made of four layers for a total of 342 atoms. All atomic positions were let to relax but the bottom gold layer. For the standing orientation, only the *fcc* adsorption site was considered.

Single-point DFT+U calculations were also computed on optimized structures within the Dudarev[7] implementation. The effective parameter $U_{\mathrm{eff}} = U - J$ was applied only to the titanium atom, where U_{eff} is the effective repulsion between electrons localized on the same site; a value of 3 eV for the

3*d* orbitals was used according to literature.[8,9] U_{eff} was introduced for obtaining more accurate Ti 3*d* energies since pure DFT functionals tend to over-delocalize the electron density.

STM images were simulated at the pDFT+U level on the optimized pDFT structure of the isolated [CpTiCot]@Au and on the monolayer array of [CpTiCot]@Au according to the Tersoff–Hamann approximation[10] as implemented in CP2K. The computed bias ranged from -2.0 V to +3 V.

Non-periodic all-electron DFT calculations on isolated molecules and clusters. These sets of calculations were performed with the ORCA 4.0 package of programs. def2-TZVP basis sets[11] were employed for Ti, C, and H, while ma-def2-TZVP with def2-ECP replacing 60 core electrons was chosen for the Au atoms.[12] The cluster models (see **Figure S9**) were obtained by a tailored cut of the previously optimized structures at the periodic DFT level. A fourth slab was added to reproduce better the surface Fermi states.[13] Thus, the isolated-molecule model was composed of one [CpTi(cot)] molecule and 40 gold atoms, [CpTi(cot)]@Au_{40}. The cluster model was used exclusively to perform all-electron calculations and have access to the Ti*2p* and C*1s* energies computed within the Koopmans' theorem framework to be compared with the experimental XPS values. For each model, single point all-electron DFT calculations were performed with two hybrid functionals (PBE0[10] and B3LYP[10]). A deviation of ca. -2% and ca. -2.5% was computed for the DFT absolute binding energy values for C *1s* and average Ti $2p_{1/2,3/2}$ with respect to our experimental data. A benchmarking study was also performed to verify the accuracy of our computational set up in determining the Ti *2p* binding energies as a function of titanium oxidation state. Only the PBE0 functional was used for these calculations. Two series of compounds were chosen, one comprising [(η-Cp^s)$_2$$Ti^{II}$], [Cp$Ti^{III}$(cot)], and [Cp$Ti^{IV}$(cht)], with Cp^s = $C_5Me_4(SiMe_2Bu^t)$[14,15], and the other containing [$Cp_2Ti^{IV}Cl_2$] and $Cp_2Ti^{III}Cl$. In the first set, the titanium coordination environment is about the same along the series, while the second set contains two Ti bent-sandwich compounds whose experimental XPS Ti *2p* data are available.[16] A difference of *ca.*1 eV was computed for Ti^{II}-Ti^{III} and Ti^{III}-Ti^{IV} for the first series against *ca.* 2 eV for the

second (see **Table S3**). When comparing the computed XPS Ti *2p* with the experimentally available data, an average error of about 3 eV was observed on the absolute energies, while an overall good agreement for the relative binding energies trend was found (2.4 eV(exp.) vs 2.4 eV(calc.)).

2 - Additional Information

2.1 – Supporting Figures and Tables

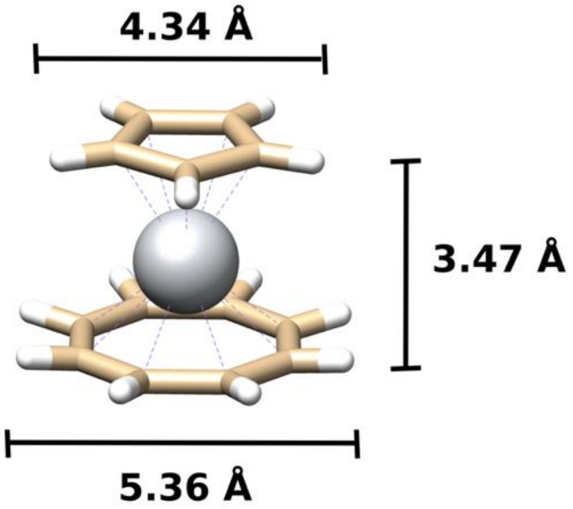

Figure S1. Structure of [CpTi(cot)] reported in Ref. S1. Atoms color code: white, hydrogen; light brown, carbon; gray, titanium.

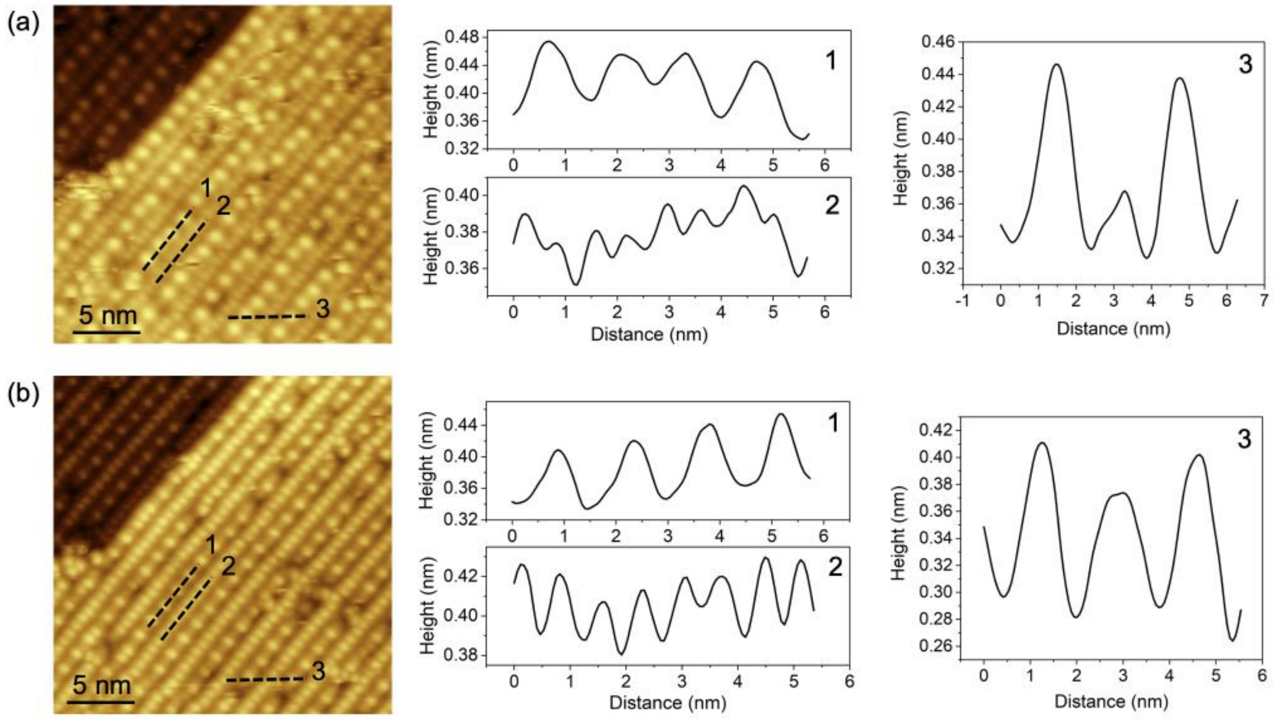

Figure S2. STM images of a monolayer of [CpTi(cot)] on Au(111) (I_t = 20pA) at +2V (a) and +1V (b). Dotted lines mark the line profiles shown on the right.

Table S1. Computed adsorption energies for different orientations of an isolated [CpTi(cot)] molecule on Au(111) adsorption sites

Orientation	Adsorption site	Adsoption energy (kcal/mol)
standing$_{cot}$	on-top	-17.0
	bridge	-18.6
	fcc	-18.7
standing$_{Cp}$	on-top	-11.7
	bridge	-12.6
	fcc	-12.8
lying	-	-13.2

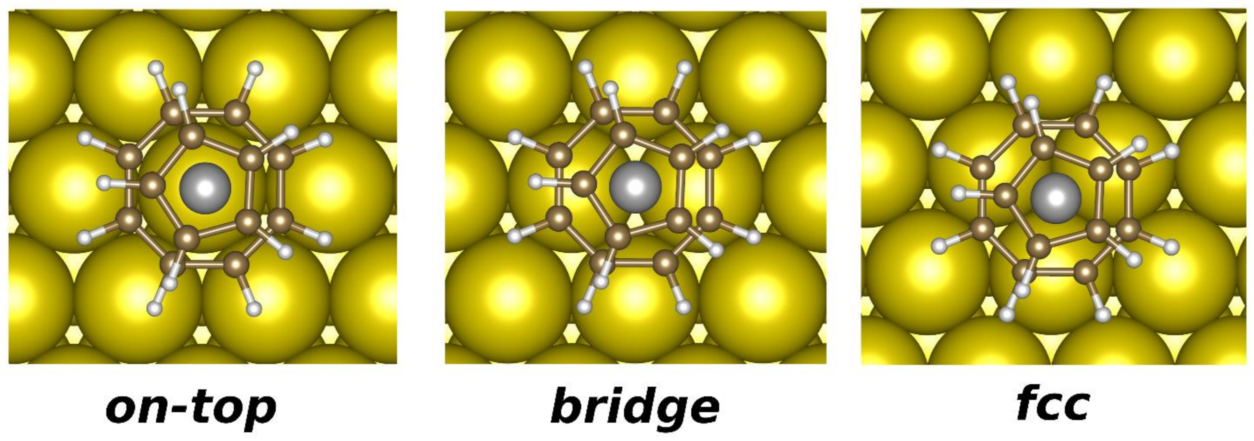

Figure S3. Different adsorption sites for a *standing*$_{cot}$ [CpTi(cot)] molecule on Au(111).

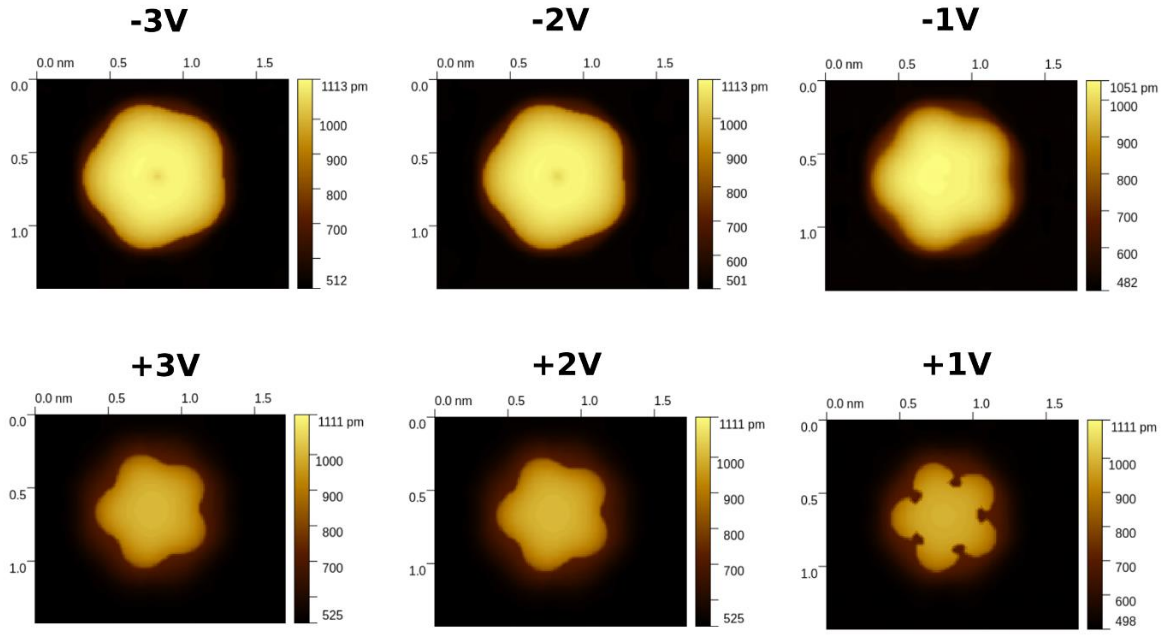

Figure S4. Simulated STM bias dependence for the *standing*$_{cot}$ adsorption arrangement.

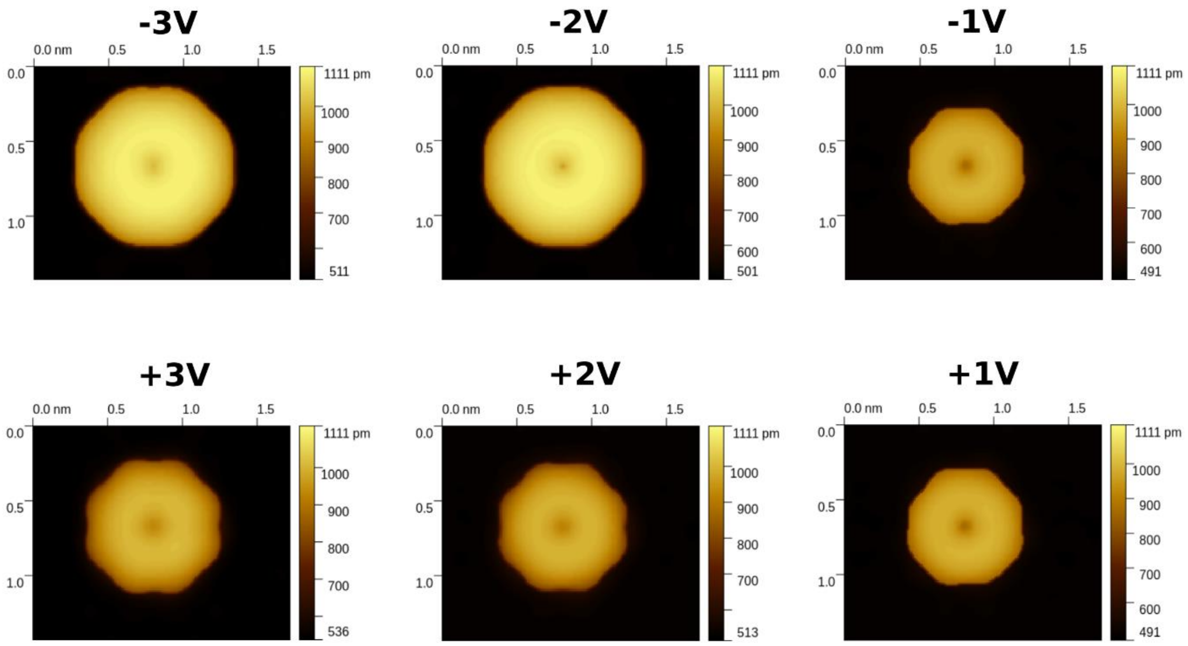

Figure S5. Simulated STM bias dependence for the *standing*$_{Cp}$ adsorption arrangement.

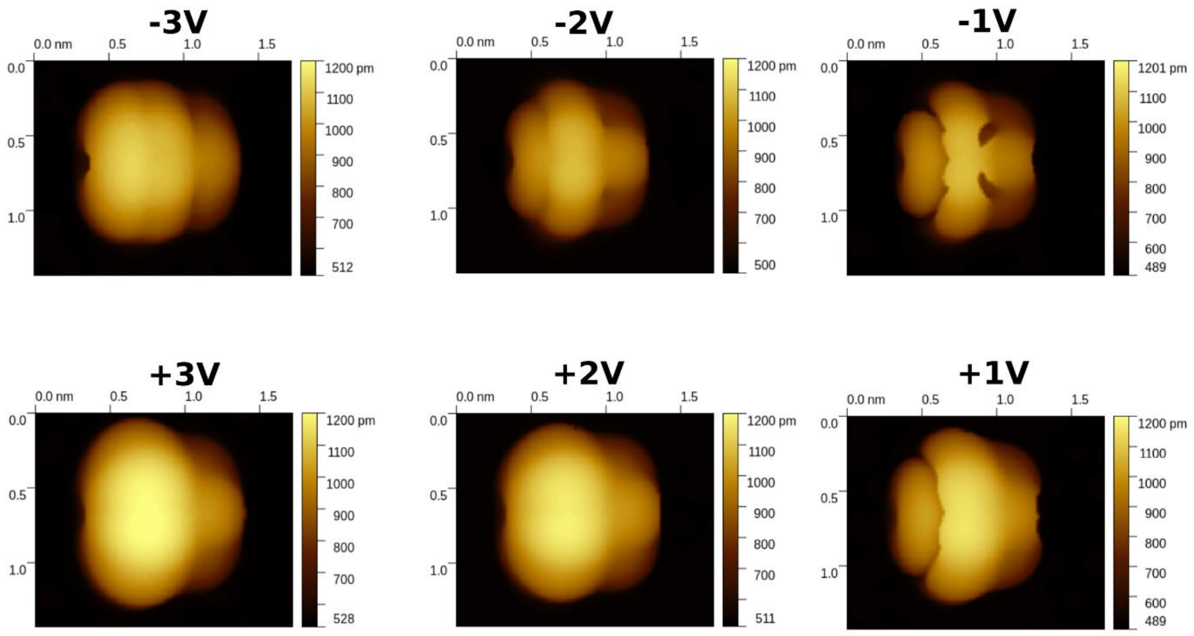

Figure S6. Simulated STM bias dependence for the *lying* adsorption arrangement.

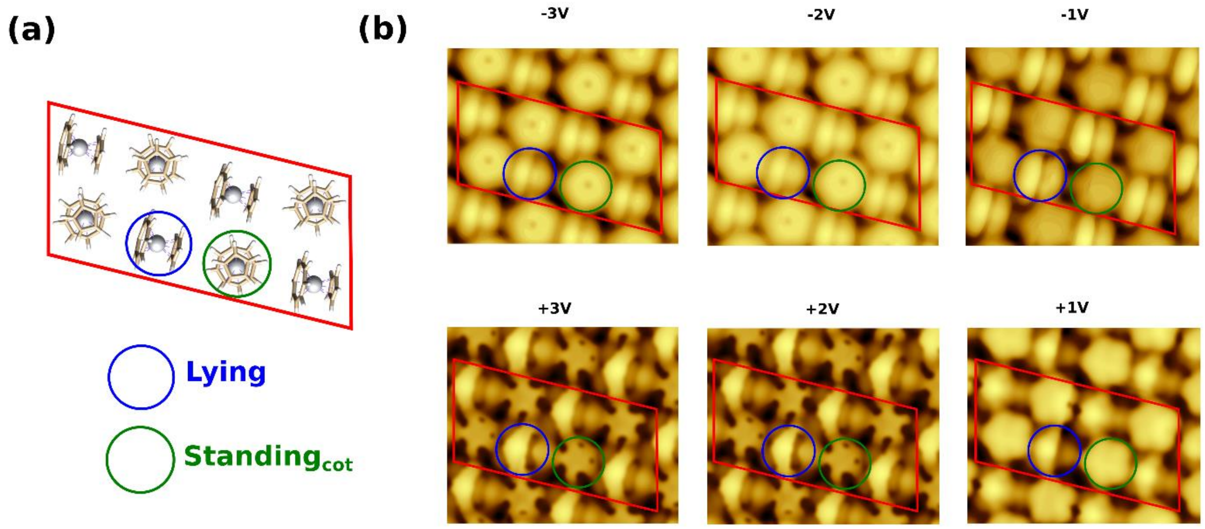

Figure S7. Simulated adsorbed monolayer with four *lying* and four $standing_{cot}$ molecules. (a) Optimized cell. (b) Simulated STM bias dependence.

Table S2. Semi-quantitative XPS elemental analysis of [CpTi(cot)] on Au(111)

	Theory	*Experiment*
**C *1s***	92.9%	94.0±4.7%
**Ti *2p***	7.1%	6.0±0.3%

Table S3. Experimental Ti $2p_{3/2}$ and computed (DFT/PBE0) Ti 2*p* binding energies (eV) for reference organometallic compounds. Data for [$Cp^s{}_2Ti$] and [CpTi(cht)] were taken from Ref. S15 and S16 respectively

Ti Oxidation State	*Compound*	*Experiment*	*Simulation*	*Compound*	*Experiment*	*Simulation*
**II**	[$Cp^s{}_2Ti$]	-	451.1	-	-	-
**III**	[CpTi(cot)]	455.2 (this work)	452.1	Cp_2TiCl	456.6	453.2
**IV**	[CpTi(cht)]	456.1	453.1	[Cp_2TiCl_2]	458.4	455.6

2.2 - UPS analysis

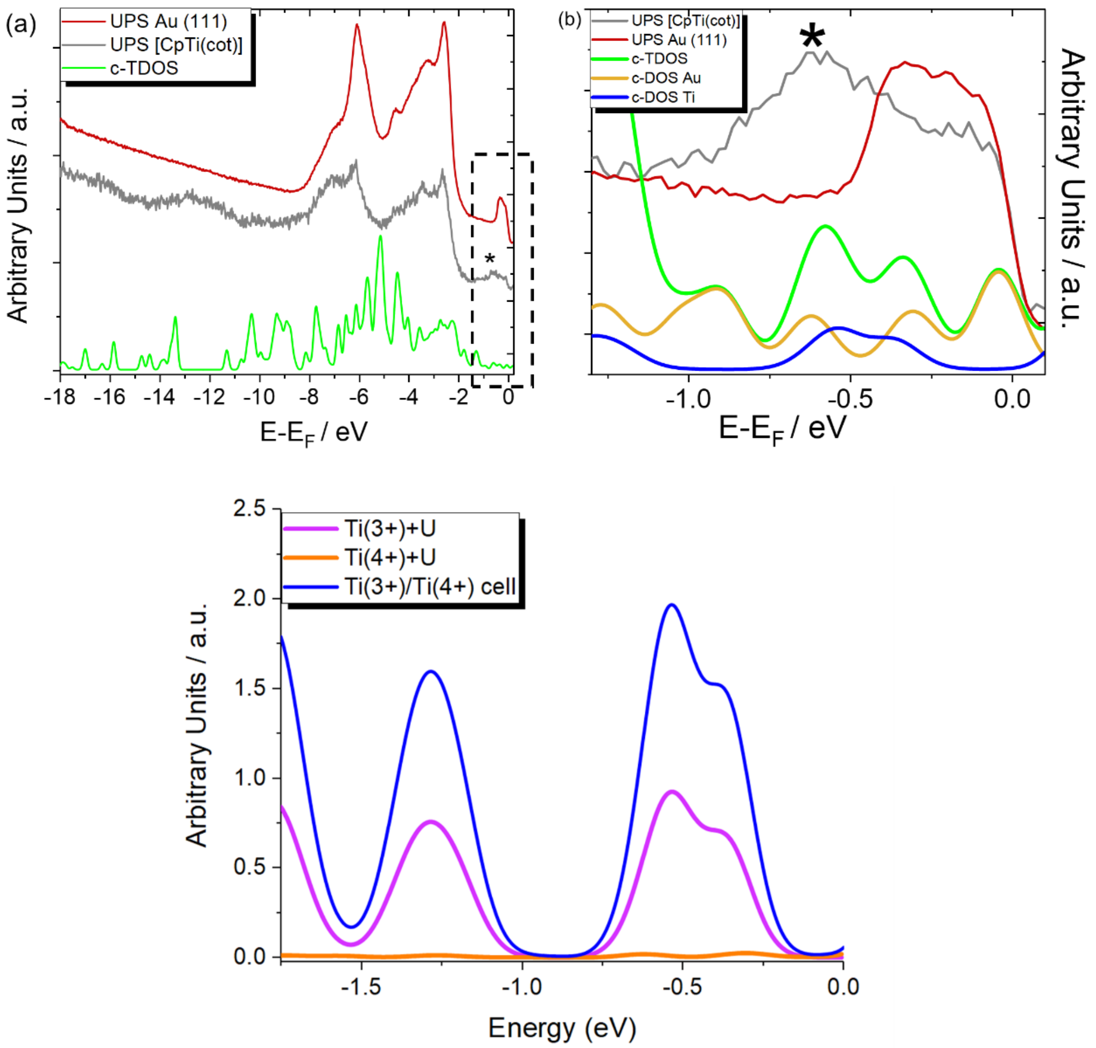

Figure S8. (a) UPS bands of [CpTi(cot)] on the Au(111) surface (gray line), c-TDOS (green line) and clean Au(111) (red line); (b) enlargement of the region between -1.75 and +0.25 eV (E-E$_F$), with the contribution of titanium and gold to the c-TDOS (blue and dark dark-yellow lines, respectively). The feature ascribable to Ti^{III} is marked with an asterisk (*); (c) contribution of Ti^{III} and Ti^{IV} to the total titanium c-DOS.

The electronic structure of the molecular film was studied experimentally by UPS and rationalized in comparison with the DOS obtained by pDFT+U on the [CpTi(cot)]@Au monolayer (**Figure S8**). A detailed description of the method employed in these calculations is reported in Ref. [17] and in the Methods section (main text). An important information on the adsorbed molecular layer is derived

from the region close to the Fermi energy, E_F, where a band around 0.8 eV appears after the [CpTi(cot)] deposition (**Figure S8b).** The comparison with the c-DOS of [CpTi(cot)] (**Figure S8b** and **c**) confirms that its presence is due to Ti^{III} in the molecular layer (marked with asterisk in **Figure S8b**), in good agreement with the DOS of other Ti^{III} systems.[18–20]

2.3 - Simulation of the XPS results

The rationalization of the four main features observed in the titanium XPS spectrum results from a comprehensive *ab initio* analysis of the electronic structure and geometric arrangement of the [CpTi(cot)] molecules in the monolayer. An alternative model for these four features assumes that the two sets of [CpTi(cot)] orientations interact differently with the gold surface but without electron transfer. In such a case, the Ti*2p* binding energies in the *lying* and *standing* dispositions would be expected to experience significantly different screening surface effects to justify the energy separation between the two (I and II) features for the Ti$2p_{3/2}$ and $2p_{1/2}$ components (2.1 eV each). Against such an interpretation, the literature reports that, for a diamagnetic analog of [CpTi(cot)], [$CpTi^{IV}$(cht)], an XPS Ti $2p_{3/2}$ signal was found at an energy only ~1 eV higher (456.1 eV).[16]

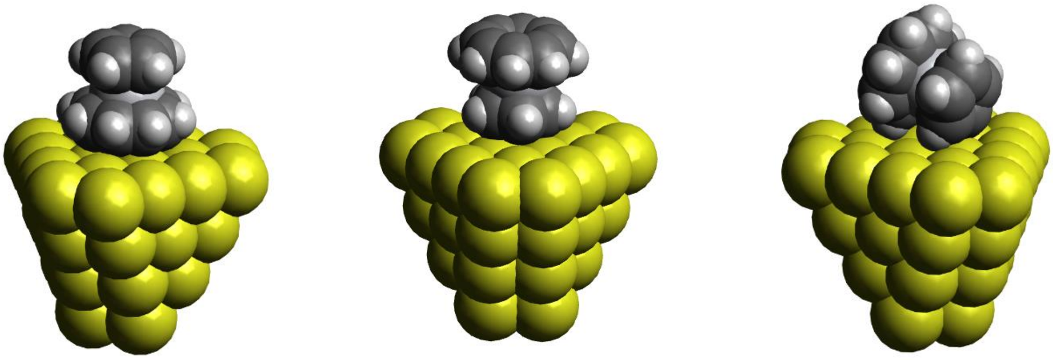

Figure S9. The three adsorption orientations of a single [CpTi(cot)] molecule adsorbed on the $Au(111)_{40}$ model employed for molecular cluster calculations: (left) *standing*$_{cot}$, (center) *standing*$_{Cp}$, and (right) *lying*.

To verify if the presence of different adsorption orientations, without any redox process involved, could be at the origin of the 455.2 eV and 457.3 eV peaks, Ti*2p* and C*1s* binding energies were also computed using a single [CpTi(cot)] molecule adsorbed on an *fcc* site of an $Au(111)_{40}$ gold cluster. The cluster model was used only to perform all-electron calculations and have access to the Ti*2p* and C*1s* energies computed within the Koopmans' theorem framework (to be compared with the experimental XPS values), since the only way to accomplish this task at the periodic level is the use of high hardware resources demanding the Gaussian augmented-plane-waves (GAPW) approach. The *fcc* optimizations, in turn, were performed in three distinct molecular dispositions at the pDFT level (see Methods and **Figure S9**). Consistent trends were found for both hybrid functionals, PBE0 and B3LYP (**Table S4 and Fig. S9**): *lying*, $standing_{Cp}$, and $standing_{cot}$ (from the lowest to the highest values of binding energy, respectively). Such a trend can be ascribed to the different screening effects of the gold surface on the carbon and titanium atoms. With regards to Ti*2p*, taking the *lying* conformation as reference, binding energies higher than 0.20(DFT) eV and 0.45(DFT) eV were found for the $standing_{Cp}$ and $standing_{cot}$, respectively. Considering the C*1s* binding energies, values were obtained averaging the results computed for each carbon atom belonging to the Cp and cot ligands. The same trend found for Ti*2p* was obtained for C*1s*. Based on the above results, the binding energies computed for the three molecular arrangements differ of an energy amount much smaller than the one experimentally observed for the features I and II of each Ti$2p_{3/2}$ and $2p_{1/2}$ component (2 eV). The same considerations are valid for C*1s*. Therefore, an *orientation-only* origin for the split I and II features can be excluded. Shake-up features along with redox processes seem the only possible explanation for the two pairs of peaks. In such a case, the peak I and its shoulder II observed for C*1s* can be rationalized as the best representation of an almost continuous distribution of C*1s* contributions due to two (at least) different molecular orientations after adsorption on Au(111) (see **Figure S10**).

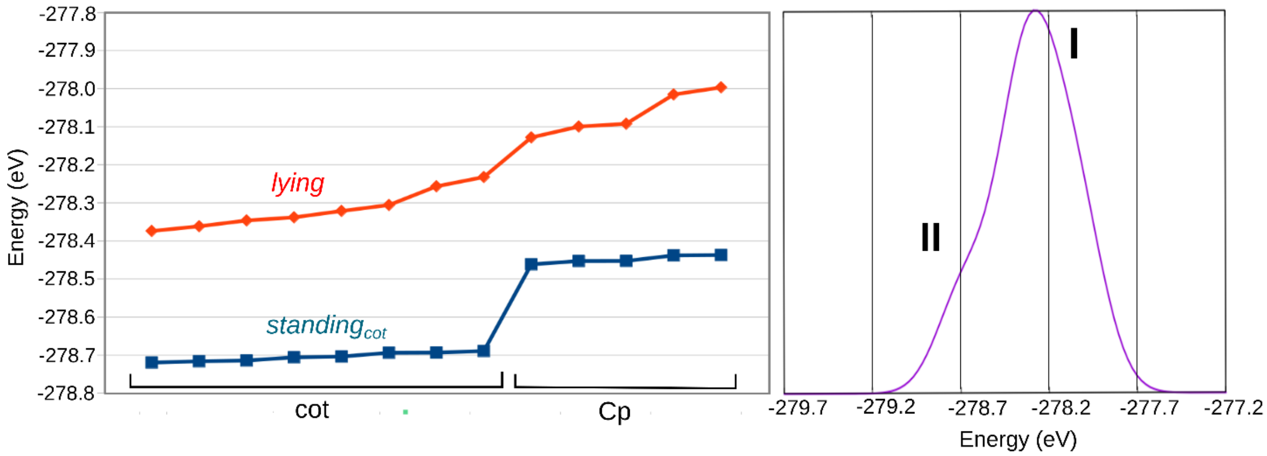

Figure S10. (left) Energies of the C *1s* orbitals computed at the DFT/PBE0 level of theory for the cluster model [CpTi(cot)]@Au(111)$_{40}$ on the *lying* and *standing*$_{cot}$ orientations. (right) Resulting C *1s* peak employing the computed energies and a Gaussian broadening of 0.15 eV.

Summarizing the previous discussion and the one presented in the main text, DFT calculations suggest that [CpTi(cot)] is unlikely to adsorb on Au(111) with a single molecular orientation. Additionally, also based on adsorption energies/geometries and XPS C*1s* spectrum, it can be proposed that at least two orientations are indeed present on the metal surface. Considering the presence of the two most stable dispositions, *lying* and *standing*$_{cot}$, the observed 2.4:1 XPS C*1s* *ratio* for the I/II features can be rationalized with a proportion of three *lying* to one *standing* molecules. Indeed, based on the computed energies reported in **Figure S10**, the 13 *standing*$_{cot}$ carbon atoms show higher binding energies than the *lying* ones. Moreover, at 485.5 eV a continuum of binding energies of Cp carbons (*standing*$_{cot}$) and cot carbons in the *lying* orientation is expected. Therefore, it is likely that all the 13 carbon atoms of the *standing*$_{cot}$ orientation (see **Figure S10**) contribute to the second feature (II) of the C*1s* XPS spectrum (**Figure 4** in the main text). On the other hand, all cot and Cp carbons of the *lying* molecules, which have lower binding energies, contribute to the simulated peak I centered at 284.8 eV. A theoretical 39:13 ratio *vs* an experimental 2.4:1 is then obtained. A unit cell containing a combination of lying and standing molecules would also agree with the reported "paired conformation" nickelocene layers adsorbed on copper or lead

single crystals.[S7,S8] According to the literature, such layers are formed by alternating vertical (Cp parallel to the surface, bright spots in the STM images) and horizontal (Cp perpendicular to the surface, dark spots) molecular arrangements on the metal.

Table S4. Computed Ti 2*p* binding energies (in eV) at different levels of theory for the different molecular dispositions in the cluster model [CpTi(cot)]@Au(111)$_{40}$

Disposition	Hartree-Fock	DFT/PBE0	DFT/B3LYP
**isolated**	484.8	452.1	450.1
standing$_{cot}$	484.7	452.3	450.4
standing$_{Cp}$	484.6	452.1	450.1
lying	484.3	451.8	449.9

2.4 – Simulated PDOS

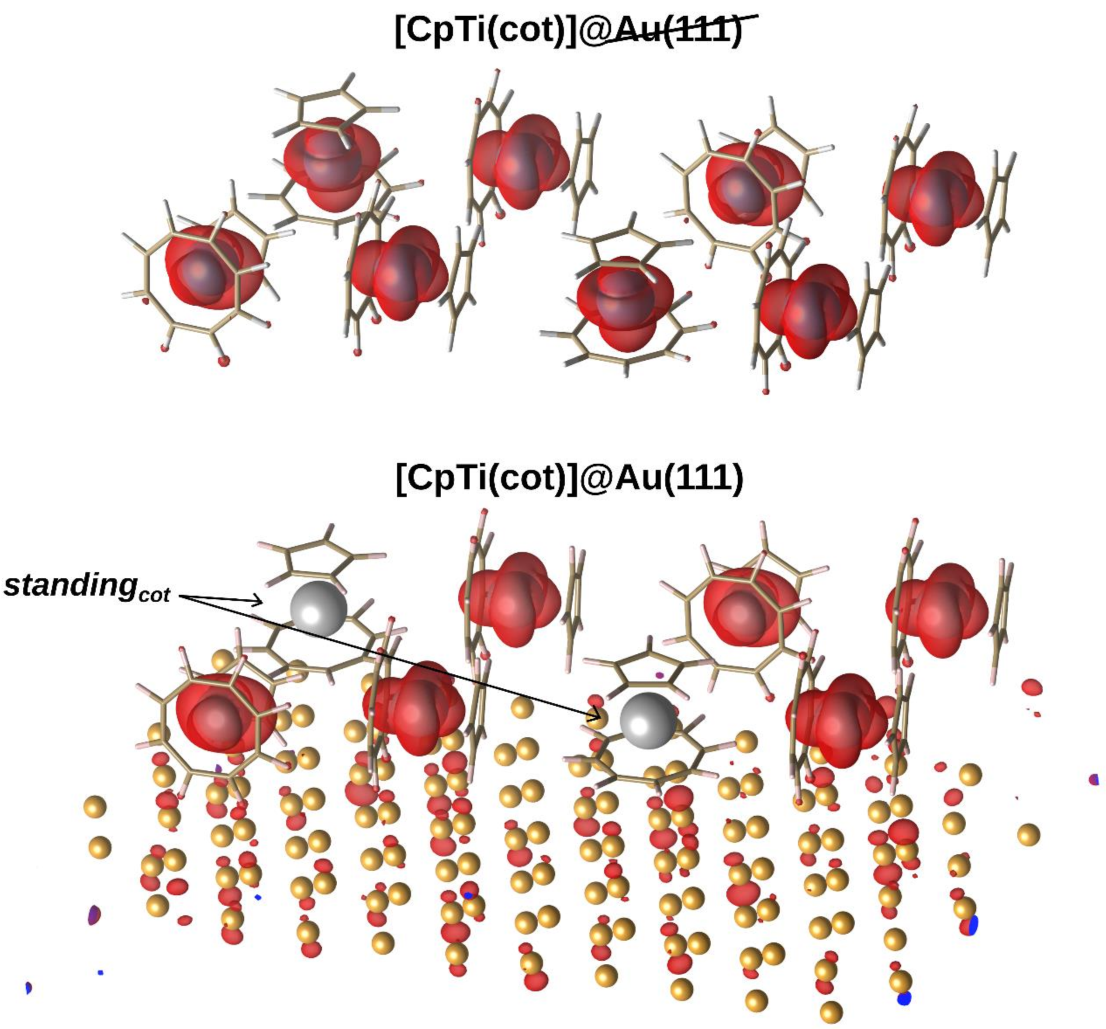

Figure S11. (up) Positive spin densities simulated when the gold surface is removed from the optimized array of the [CpTi(cot)]@Au monolayer. (down) Simulated positive spin densities including the gold surface in [CpTi(cot)]@Au. The isosurfaces are red colored and are drawn for a value of 0.001 e bohr^{-3}.

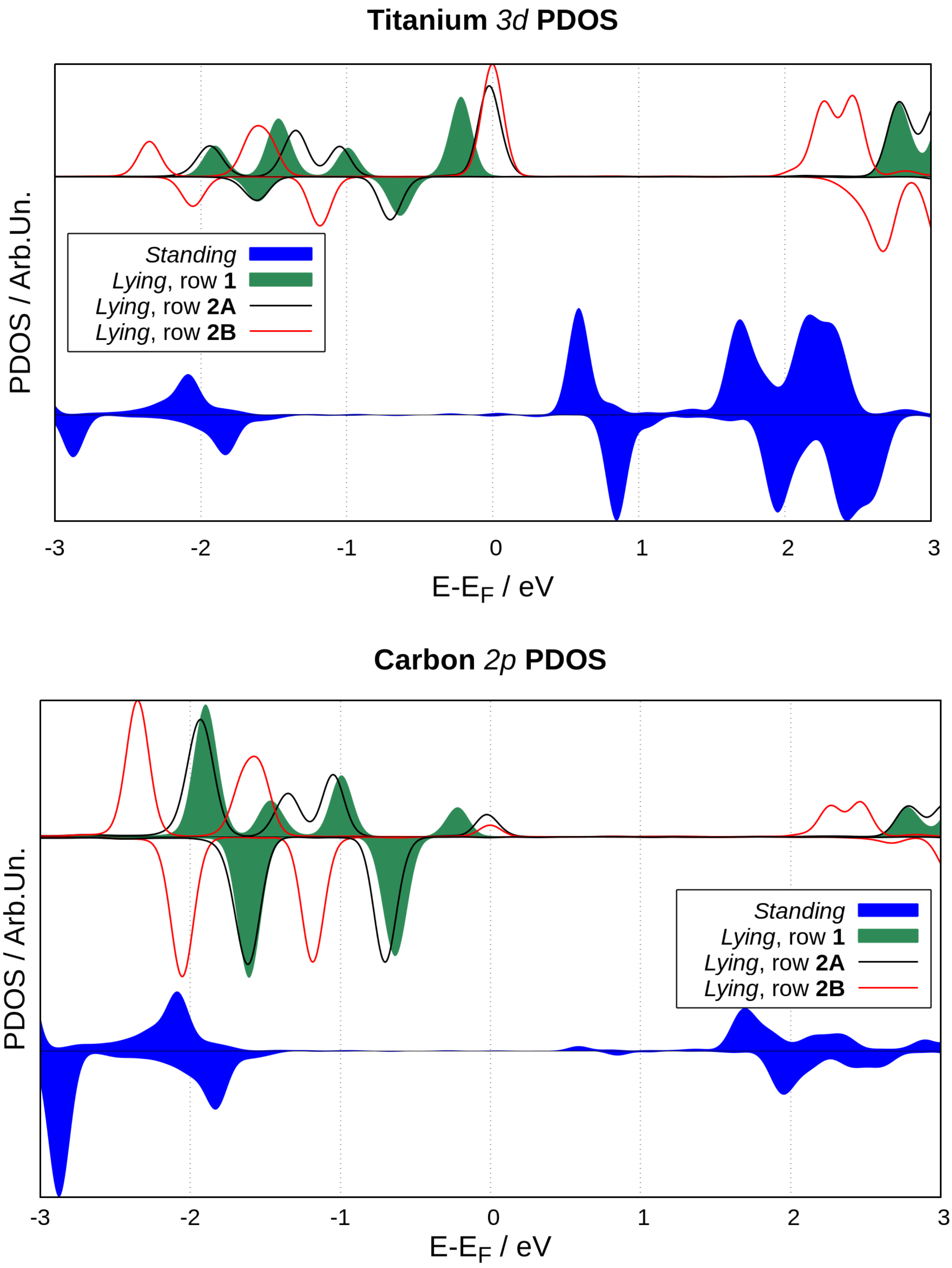

Figure S12. Partial Density of States (PDOS) for the Ti *3d* (top panel) and C *2p* (bottom) orbital components comprising 3 eV below and above the Fermi energy and considering the different types of molecules (*standing* and *lying*) inside the monolayer unit cell.

The Ti *3d* orbitals of the *lying* molecules mainly contribute to the PDOS at negative biases, while those from the *standing* molecules appear at positive ones. The major contributions of the Ti *3d* states are observed at higher energies (close to the Fermi level) than the negative biases experimentally used in STM experiments. Noticeably, while both at -2 eV and -1 eV minor *3d* components are observed for the *lying* molecules, those related to the *standing* molecules are seen only at -2 eV. This agrees with the simulations presented in Figure 3, where the *standing* molecules show brighter spots at the bias of -2 V than at -1 V. For positive bias, only contributions from the *standing* molecules are observed up to +2 V. In the simulated STM images, however, rows 2 are not bright for biases below +2 V. This is because, in the *standing* orientation, the carbon electron density of the ligand shields the *3d* orbitals from the STM tip. As a consequence, their contributions are not as relevant as those from the *lying* molecules, where the metal ion is directly accessible to the tip.

The C *2p* PDOS support the above statements: a non-negligible spin polarization is present for all types of molecules and, despite an overall similarity with the *3d* component, the *standing* molecules do not show any significant contribution from -1.5 to +1.5 eV, at variance from the *standing* Ti *3d* components. Conversely, the *lying* molecules cover the energy window from -1.5 to the Fermi energy, while the empty states are available only above +2 eV. The availability of empty *2p* states at different energies for the *standing* and *lying* molecules explains why we observe an upshift in the calculated STM profiles with respect to the experimental positive bias.

We can therefore state that, due to the different orbitals exposed to the STM tip as a function of the molecular orientation, i) both the Ti *3d* and C *2p* orbitals contribute to the on/off bias dependence shown by the *lying* molecules; ii) only the C *2p* orbitals contribute to the observed bias dependence presented by the *standing* molecules.

References

(1) Camargo, L. C.; Briganti, M.; Santana, F. S.; Stinghen, D.; Ribeiro, R. R.; Nunes, G. G.; Soares, J. F.; Salvadori, E.; Chiesa, M.; Benci, S.; Torre, R.; Sorace, L.; Totti, F.; Sessoli, R. Exploring the Organometallic Route to Molecular Spin Qubits: The [CpTi(Cot)] Case. *Angew. Chemie Int. Ed.* **2021**, *60* (5), 2588–2593. https://doi.org/10.1002/anie.202009634.

(2) Perdew, J. P.; Burke, K.; Ernzerhof, M. Generalized Gradient Approximation Made Simple. *Phys. Rev. Lett.* **1996**, *77* (18), 3865–3868. https://doi.org/10.1103/PhysRevLett.77.3865.

(3) Zhang, Y.; Yang, W. Comment on "Generalized Gradient Approximation Made Simple." *Phys. Rev. Lett.* **1998**, *80* (4), 890–890. https://doi.org/10.1103/PhysRevLett.80.890.

(4) Kraus, S.; Herman, A.; Huttmann, F.; Bianchi, M.; Stan, R. M.; Holt, A. J.; Tsukamoto, S.; Rothenbach, N.; Ollefs, K.; Dreiser, J.; Bischof, K.; Wende, H.; Hofmann, P.; Atodiresei, N.; Michely, T. Uniaxially Aligned 1D Sandwich-Molecular Wires: Electronic Structure and Magnetism. *J. Phys. Chem. C* **2022**, *126* (6), 3140–3150. https://doi.org/10.1021/acs.jpcc.1c10625.

(5) Sabatini, R.; Gorni, T.; De Gironcoli, S. Nonlocal van Der Waals Density Functional Made Simple and Efficient. *Phys. Rev. B - Condens. Matter Mater. Phys.* **2013**, *87* (4), 4–7. https://doi.org/10.1103/PhysRevB.87.041108.

(6) Goedecker, S.; Teter, M.; Hutter, J. Separable Dual-Space Gaussian Pseudopotentials. *Phys. Rev. B* **1996**, *54* (3), 1703–1710. https://doi.org/10.1103/PhysRevB.54.1703.

(7) Dudarev, S. L.; Botton, G. A.; Savrasov, S. Y.; Humphreys, C. J.; Sutton, A. P. Electron-Energy-Loss Spectra and the Structural Stability of Nickel Oxide: An LSDA+U Study. *Phys. Rev. B* **1998**, *57* (3), 1505–1509. https://doi.org/10.1103/PhysRevB.57.1505.

(8) Hu, Z.; Metiu, H. Choice of U for DFT+ U Calculations for Titanium Oxides. *J. Phys. Chem. C* **2011**, *115* (13), 5841–5845. https://doi.org/10.1021/jp111350u.

(9) Capdevila-Cortada, M.; Łodziana, Z.; López, N. Performance of DFT+ U Approaches in the Study of Catalytic Materials. *ACS Catal.* **2016**, *6* (12), 8370–8379. https://doi.org/10.1021/acscatal.6b01907.

(10) Tersoff, J.; Hamann, D. R. Theory of the Scanning Tunneling Microscope. *Phys. Rev. B* **1985**, *31* (2), 805–813. https://doi.org/10.1103/PhysRevB.31.805.

(11) Weigend, F.; Ahlrichs, R. Balanced Basis Sets of Split Valence, Triple Zeta Valence and Quadruple Zeta Valence Quality for H to Rn: Design and Assessment of Accuracy. *Phys. Chem. Chem. Phys.* **2005**, *7* (18), 3297. https://doi.org/10.1039/b508541a.

(12) Andrae, D.; Häußermann, U.; Dolg, M.; Stoll, H.; Preuß, H. Energy-Adjusted Ab Initio Pseudopotentials for the Second and Third Row Transition Elements. *Theor. Chim. Acta* **1990**, *77* (2), 123–141. https://doi.org/10.1007/BF01114537.

(13) Lunghi, A.; Iannuzzi, M.; Sessoli, R.; Totti, F. Single Molecule Magnets Grafted on Gold: Magnetic Properties from Ab Initio Molecular Dynamics. *J. Mater. Chem. C* **2015**, *3* (28), 7294–7304. https://doi.org/10.1039/C5TC00394F.

(14) Luo, Y.; Ohno, K. Computational Study of Titanocene-Catalyzed Dehydrocoupling of the

Adduct $Me_2NH{\cdot}BH_3$: An Intramolecular, Stepwise Mechanism. *Organometallics* **2007**, *26* (14), 3597–3600. https://doi.org/10.1021/om7003892.

(15) Hitchcock, P. B.; Kerton, F. M.; Lawless, G. A. The Elusive Titanocene. *J. Am. Chem. Soc.* **1998**, *120* (39), 10264–10265. https://doi.org/10.1021/ja981934e.

(16) Groenenboom, C. J.; Sawatzky, G.; de Liefde Meijer, H. J.; Jellinek, F. Electron Spectroscopy of Some Cyclopentadienylcycloheptatrienylmetal Compounds. *J. Organomet. Chem.* **1974**, *76* (1), C4–C6. https://doi.org/10.1016/S0022-328X(00)90324-3.

(17) Lanzilotto, V.; Malavolti, L.; Ninova, S.; Cimatti, I.; Poggini, L.; Cortigiani, B.; Mannini, M.; Totti, F.; Cornia, A.; Sessoli, R. The Challenge of Thermal Deposition of Coordination Compounds: Insight into the Case of an Fe_4 Single Molecule Magnet. *Chem. Mater.* **2016**, *28* (21), 7693–7702. https://doi.org/10.1021/acs.chemmater.6b02696.

(18) Tait, R. H.; Kasowski, R. V. Ultraviolet Photoemission and Low-Energy-Electron Diffraction Studies of TiO_2 (Rutile) (001) and (110) Surfaces. *Phys. Rev. B* **1979**, *20* (12), 5178–5191. https://doi.org/10.1103/PhysRevB.20.5178.

(19) Henrich, V. E.; Dresselhaus, G.; Zeiger, H. J. Observation of Two-Dimensional Phases Associated with Defect States on the Surface of TiO_2. *Phys. Rev. Lett.* **1976**, *36* (22), 1335–1339. https://doi.org/10.1103/PhysRevLett.36.1335.

(20) Chung, Y. W.; Lo, W. J.; Somorjai, G. A. Low Energy Electron Diffraction and Electron Spectroscopy Studies of the Clean (110) and (100) Titanium Dioxide (Rutile) Crystal Surfaces. *Surf. Sci.* **1977**, *64* (2), 588–602. https://doi.org/10.1016/0039-6028(77)90064-4.
